# Pulmonary intralobar sequestration associated with aspergillosis and aspiration

**DOI:** 10.1002/ccr3.3039

**Published:** 2020-06-30

**Authors:** Daniel J. Zaccarini, Ola El‐Zammar

**Affiliations:** ^1^ Pathology SUNY Upstate University Hospital Syracuse NY USA

**Keywords:** aspergillosis, aspiration, bronchopulmonary sequestration

## Abstract

The pathogenesis of intralobar pulmonary sequestration is not completely understood, either representing a congenital or acquired process. The presence of fungal organisms and aspiration has both clinical and etiologic significance.

## Graphical abstract

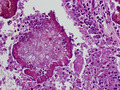

## 1 INTRODUCTION

Bronchopulmonary sequestration accounts for a portion of cystic anomalies of the lung that, by definition, have an arterial supply. We describe a case report of a young female presenting with intralobar sequestration associated with aspergillosis. There have been 38 prior cases, and none report additional findings of aspiration.

Benign cystic congenital anomalies of the lung include congenital pulmonary airway malformation (CPAM) and bronchopulmonary sequestration (BPS). BPS is less common than CPAM and is divided into intralobar and extralobar types. By definition, BPS should have a systemic arterial supply and usually lacks connection with the bronchial airways^1^ (Table 1). The arterial supply is usually from the thoracic aorta; however, it less often originates from the abdominal aorta, intercostal, celiac, subclavian, and internal mammary vessels.^2^, ^3^ In contrast, CPAM should not have a systemic blood supply and typically shows connection to the bronchopulmonary tree. Additionally, CPAM typically occurs in neonates and less commonly in adults.^4^

**TABLE 1:** Benign cystic congenital lung anomalies

Benign cystic congenital lung anomalies	Intralobar sequestration	Extralobar sequestration	Congenital pulmonary airway malformation
Systemic blood supply	Present	Present	Not present
Age	Infants and adults	Neonates and infants	Typically neonates, less common in adults
Typical location	Left lower lobe	Lower thoracic cavity with unique separate pleura	Depends on subtype

Bronchopulmonary sequestration is divided into intralobar and extralobar sequestration, whereas the former occurs in a normal lung lobe, while the latter contains a unique separate pleura.^5^ Intralobar sequestration typically occurs more often in the left lung, classically the lower lobe. Intralobar sequestration seems to equally affect infants and adults, while extralobar sequestration is typically seen in neonates and infants. Congenital diseases such as gastrointestinal duplication, congenital diaphragmatic hernia, accessory spleen, cardiovascular diseases (total anomalous pulmonary venous return and truncus arteriosus), and pulmonary hypoplasia have been seen associated with extralobar sequestration.^6^ Associated anomalies are less commonly reported with intralobar sequestration and include esophagobronchial diverticula and fistulas, and skeletal anomalies (mostly scoliosis).^6^ It is important to recognize and properly treat BPS because infrequent complications can occur such as fatal bleeding.^7^ In addition, squamous cell carcinoma has been scarcely reported to arise within an intralobar sequestration.^8^

The purpose of this case report is to describe a rare association of aspergillosis and intralobar sequestration and the importance of pathologic examination to assess for aspiration.

## 2 CASE PRESENTATION

**FIGURE 1:**
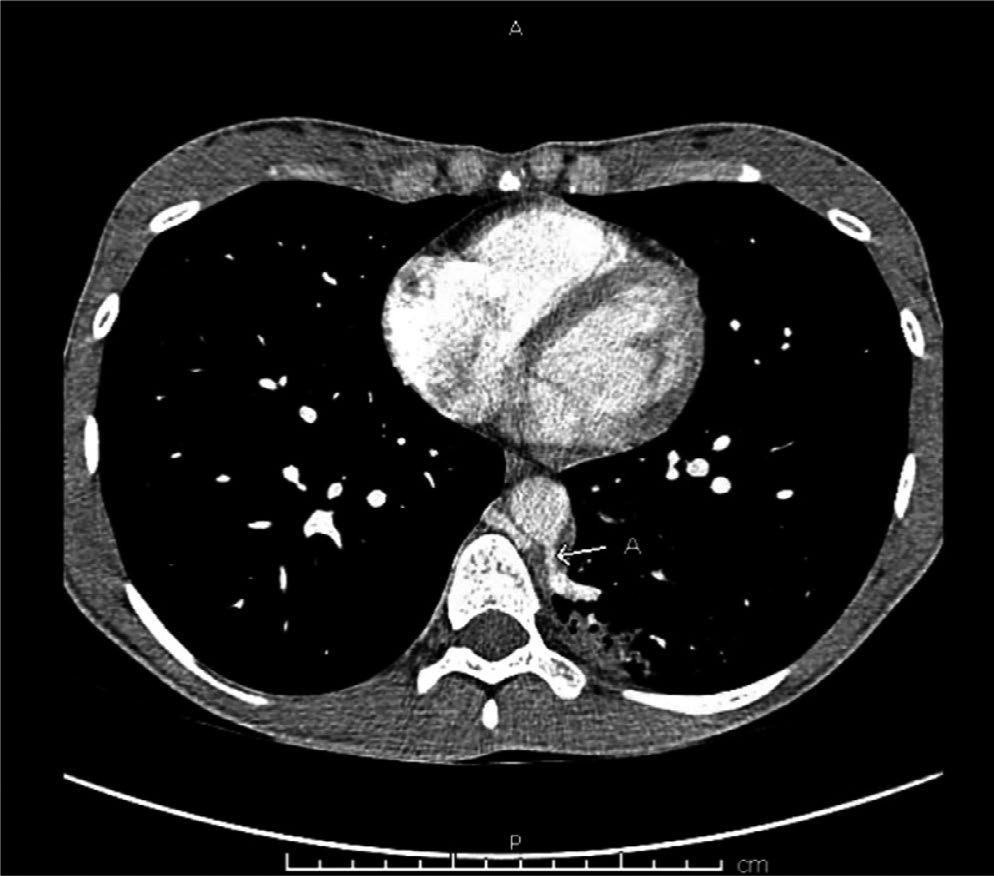
Computer tomography thorax with contrast (mediastinal window) showing cystic structure in superior segment of left lower lobe. Anomalous branches from the thoracic aorta extent to the cystic structure (arrow)

An 18‐year‐old girl presented with recurrent lung infections for which she had been receiving antibiotics. The patient also had a history of asthma, and there was no reported history of aspiration. Computer tomography (CT) demonstrated findings suggestive of a sequestration with a direct arterial blood supply from the aorta and direct venous drainage from the azygos vein (Figure 1). The lesion was located in the lower lobe basal segments, and a connection to the tracheobronchial tree was not seen. A segmentectomy was performed, and intraoperatively, there was a large left lower lobe pulmonary sequestration that had a three‐vessel blood supply directly from the aorta, as well as to venous tributaries going to the azygos vein. Otherwise, there was no other pathology that was noted in the chest. When performing the segmentectomy, it was noted that there were small basilar segmental bronchi that were transected.

**FIGURE 2:**
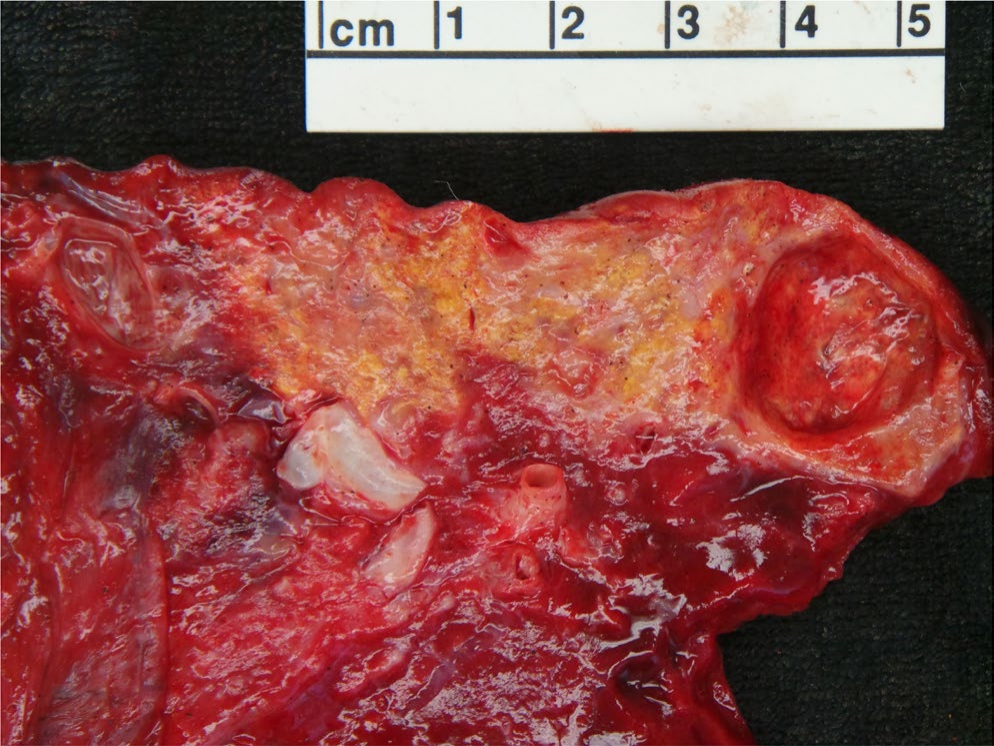
Gross photograph shows the sequestration with a yellow‐tan area and cystic change

**FIGURE 3:**
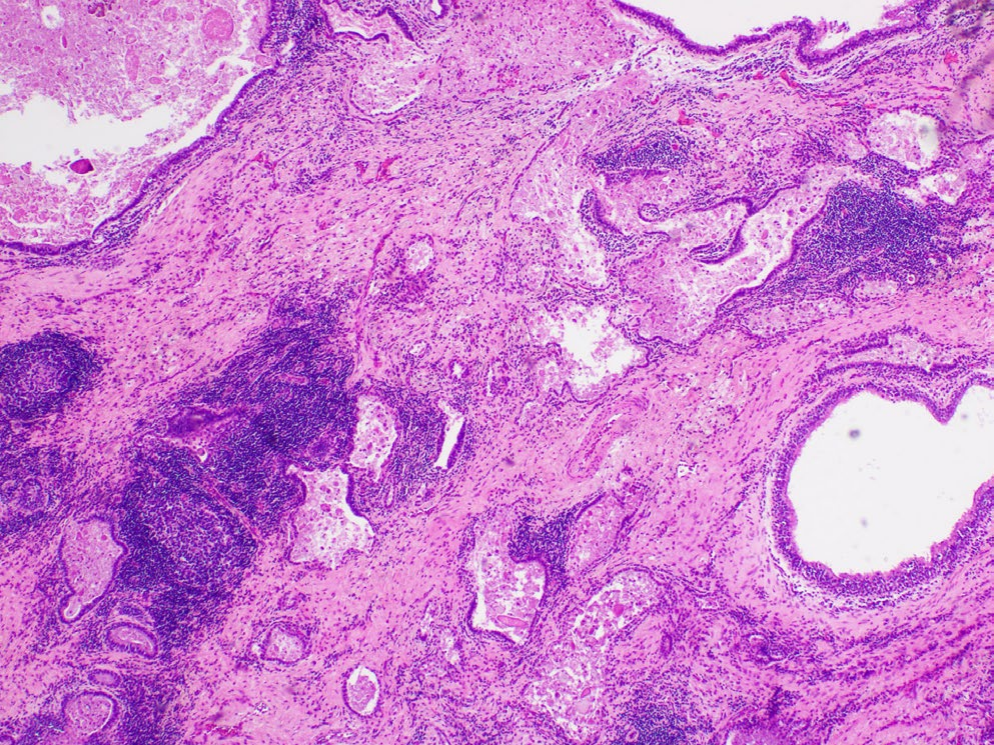
Cystic lesion showing chronic inflammation and fibrosis

**FIGURE 4:**
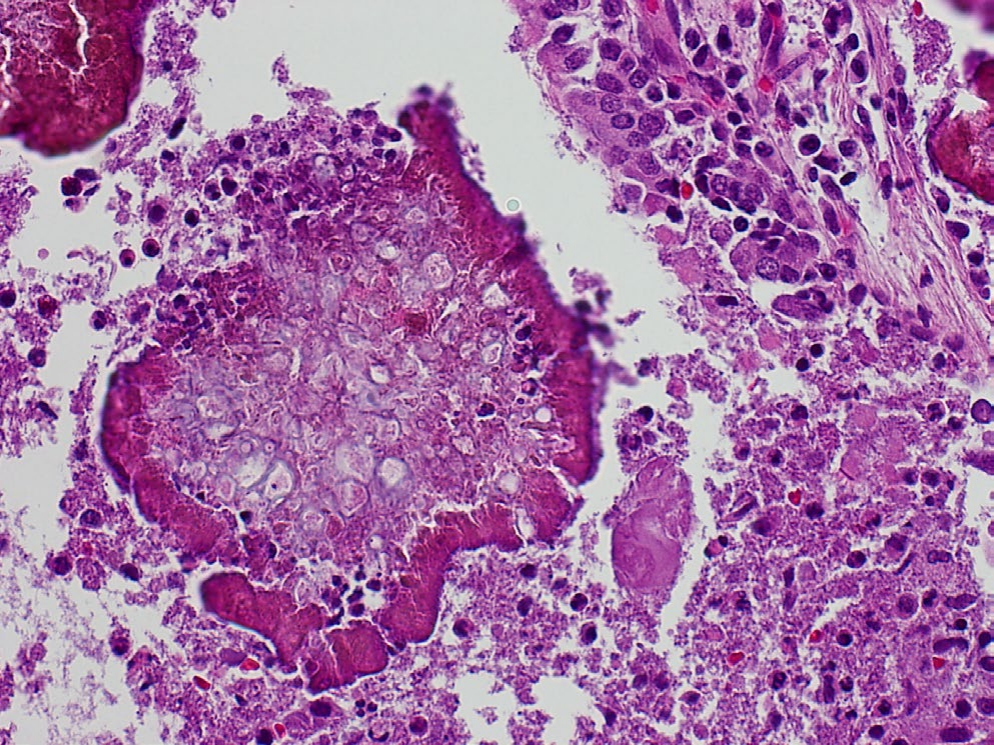
High‐power view shows fungal organisms centrally surrounded by Splendore–Hoeppli phenomenon

**FIGURE 5:**
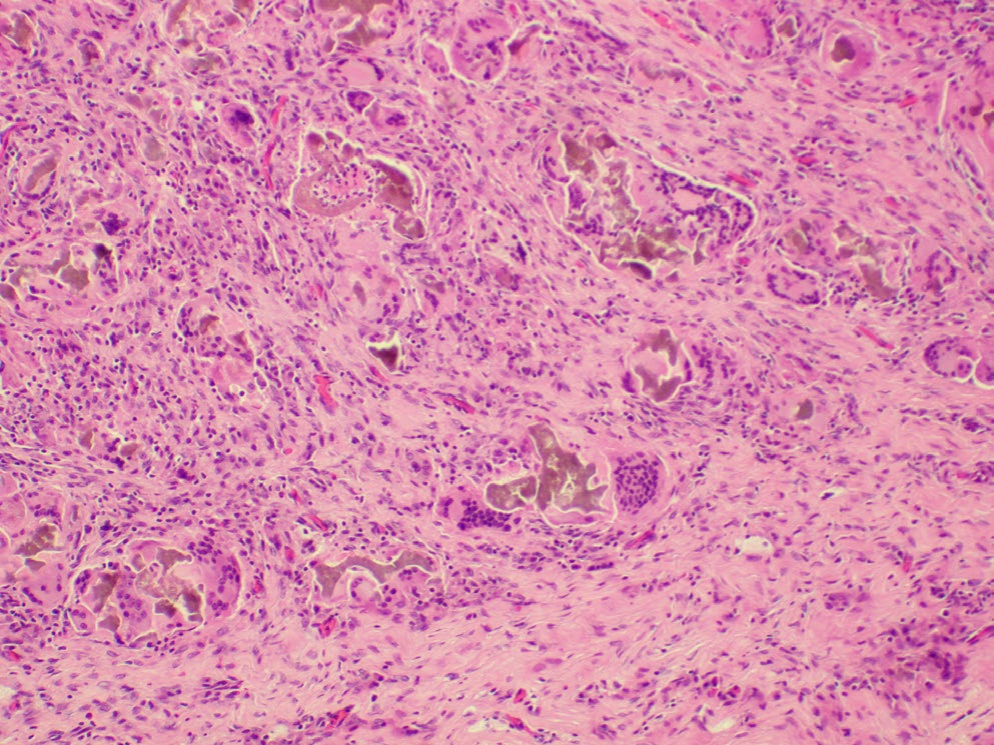
Degenerated pigmented material is surrounded by a giant cell reaction

The segment of lung parenchyma grossly demonstrated yellow‐tan areas and cystic change (Figure 2). Microscopic findings demonstrated lung parenchyma with fibrosis and chronic inflammation (Figure 3). Fungal structures were seen within airspaces and embedded in amorphous eosinophilic material that resembles Splendore–Hoeppli phenomenon (Figure 4). Gram stain and acid‐fast bacilli—Fite (AFB‐Fite) were negative. Gomori methenamine silver (GMS) highlighted hyphae with branching that would be compatible with Aspergillus. There was no evidence of invasion into lung parenchyma. Additionally, degenerated pigmented‐to‐opaque material was seen within airspaces and surrounded by a giant cell reaction, and this was suggestive of aspiration (Figure 5). Furthermore, the smaller vessels demonstrated intimal and medial thickening, a feature that is seen in pulmonary sequestration. The patient was not treated for a fungal infection and was asymptomatic at follow‐up. However, barium swallow was performed to assess for aspiration and revealed flash penetration of thin liquid. The patient is being followed for pharyngeal dysphagia, and the workup for the cause is ongoing.

## 3 DISCUSSION

Although most intralobar sequestrations do not have a connection to the bronchopulmonary airways, it is reported that around 15% of cases may show a connection.^2^ In addition to being connected to the bronchi, the sequestration can rarely be connected to the lung parenchyma or the gastrointestinal tract.^1^, ^9^ The term "congenital bronchopulmonary foregut malformation" has been suggested by Gerle because of these less common findings.^10^ Aspergillus fungal organisms have been reported in intralobar sequestrations in the past with 38 reported in the literature.^11^, ^12^, ^13^, ^14^, ^15^, ^16^, ^17^, ^18^, ^19^, ^20^, ^21^ Considering that the patient had a history of lung infections, it is possible that the Aspergillus organisms represent a superimposed infection in this case. The fact that fungal organisms have been reported in these prior cases suggests a connection to the tracheobronchial tree and possibly an acquired etiology.

The antecedent cases of aspergillosis associated with intralobar sequestration showed mostly noninvasive infection, while one case showed an acute presentation with fungal invasion.^21^ A unique aspect about the current case is that Splendore–Hoeppli phenomenon has only been reported in one other case.^11^ Additionally, the pathology demonstrated brown opaque material surrounded by a giant cell reaction which raised the possibility of aspiration, and this was mentioned in our pathology report. The subsequent barium swallow showed flash penetration of thin liquid, and the patient is being followed for pharyngeal dysphagia. None of the previous reported cases of intralobar sequestration associated with Aspergillus also had foreign material suggestive of aspiration, although there are case reports of sequestration occurring with aspiration. In this case, it was difficult to definitively rule out a component of aspiration considering the histologic findings, even though a clinical history of aspiration was not reported. A case of intralobar "pseudosequestration" has been described, where a child aspirated a plastic pen top, and subsequently demonstrated findings of chronic pneumonia and botryomycosis; however, there seemed to be no systemic feeding vessel in this report.^22^ Another case reported an infant who aspirated a juniper tree twig.^23^ A report of bilateral intrapulmonary sequestration showed communication between each sequestration and a bronchoesophageal fistula communicating with the left sequestration.^24^ Another unique aspect of this case is that the sequestration had venous drainage to the azygos vein. Most intralobar sequestrations are drained by pulmonary veins; however, other channels have been reported.^25^ About 5% drain into the azygos, hemiazygos, intercostal, or superior vena cava veins.^26^, ^27^

Grossly intralobar sequestration shows areas of cystic change and compact lung parenchyma.^26^ Pathologic findings for intralobar sequestration include hypertensive changes in vessels, fibrosis, chronic inflammation, and a relatively large feeding vessel.^5^ The histologic features of extralobar sequestration may have features similar to CPAM such as bronchiole‐like proliferations.^5^, ^28^

The pathogenesis of intrapulmonary sequestration is not fully agreed upon and, however, may occur early in lung development before separation occurs from the systemic blood supply. A study performed by Stocker and Malczak demonstrated systemic arteries arising from the thoracic aorta and traversing the pulmonary ligament in ten out of eleven random autopsy cases without congenital pulmonary or vascular disease.^23^ However, another theory is that it is related to recurrent infections and parasitization of arteries from systemic feeding vessels.^23^ Reasons why intralobar sequestration seems to be an acquired phenomenon include that it is uncommonly seen with other congenital malformations, its most common location (left lower lobe) is in close proximity to the normal pulmonary ligament arteries and lastly the usual clinical history of infection in many cases.^2^ Symptomatic lung sequestration is typically treated with resection, and outcome is generally good.

## CONFLICTS OF INTEREST

We have no financial disclosures or conflicts of interest and have not received any grant for this work.

## AUTHOR CONTRIBUTIONS

DJ.Z: contributed to write‐up of abstract, introduction, case presentation, discussion, and images. OE‐Z: reviewed and edited the manuscript.
